# Hurry Up and Wait: Timelines and Takeaways from the Biomarker Qualification Program

**DOI:** 10.1007/s43441-025-00889-6

**Published:** 2025-10-26

**Authors:** Grace Collins, Jeff D. Allen, Hillary S. Andrews, Bernat Navarro-Serer, Mark D. Stewart

**Affiliations:** Friends of Cancer Research, 1800 M Street NW, Suite 1050 South, Washington, DC USA

**Keywords:** Oncology, Biomarker, Endpoint, Qualification, FDA

## Abstract

**Background:**

The Biomarker Qualification Program (BQP), formally established in 2016 under the *21st Century Cures Act*, is a key pathway for developing novel biomarkers for regulatory use. We evaluated eight years of BQP experience to assess whether it has facilitated the qualification of novel biomarkers.

**Methods:**

We collected characteristics and submission dates for accepted biomarker qualification projects from the FDA’s Drug Development Tool Qualification Project Search database.

**Results:**

As of July 1, 2025, 61 projects were accepted into the BQP. Safety (30%), Diagnostic (21%), and PD Response (20%) biomarkers were the most common. Projects primarily used molecular (46%) and radiologic/imaging (39%) methods and were split between measures of a disease/condition or drug response/effect of exposure. Few projects included surrogate endpoint biomarkers (*n* = 5). Half of the accepted projects remained at the initial Letter of Intent (LOI) stage, and only eight biomarkers were qualified through the program. LOI and Qualification Plan (QP) reviews frequently exceeded FDA targets by three months and seven months, respectively. For projects reaching the QP stage, QP development took a median of 32 months, with surrogate endpoints taking 47 months.

**Conclusion:**

The BQP supports the development of certain biomarkers but has seen limited use for biomarkers intended as surrogate endpoints. Coupled with longer timelines for their QP development, these trends demonstrate the program may not be well-suited for advancing novel response biomarkers. Given significant stakeholder interest in novel surrogate measures, a dedicated program may better support novel response biomarker development, particularly for biomarkers with applicability across multiple drug development programs.

## Introduction

Novel biomarkers are increasingly valuable tools for accelerating evidence generation and regulatory decision-making by informing patient selection and providing earlier measures of treatment efficacy and safety. Currently, the U.S. Food and Drug Administration (FDA) offers two primary “pathways” for stakeholders seeking to develop and validate novel biomarkers for regulatory use, both of which require extensive evidence generation for a novel biomarker to be considered reliable for regulatory use. Most commonly, novel biomarker development occurs during a clinical development program, with validation through regulatory review and approval of a new drug application (NDA) or biologics license application (BLA). The second “pathway”, the Biomarker Qualification Program (BQP), began in 2007 and its review framework was formally established under the *21st Century Cures Act* on December 13, 2016, in response to stakeholder calls for a more collaborative, structured, and transparent process for biomarker development and validation [1, 2]. The BQP’s goals are to “support outreach to stakeholders for the identification and development of new biomarkers; provide a framework for the review of biomarkers for use in regulatory decision-making; and qualify biomarkers for specific contexts of use that address specified drug development needs”[3].

**Fig. 1:**
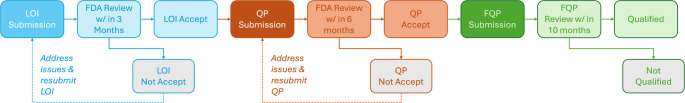
Overview of the drug development tool qualification process. The *21st Century Cures Act* established a structured, three-stage process for drug development tool qualification to provide a transparent, collaborative pathway to qualification. LOI, Letter of Intent; FQP, Full Qualification Package; QP, Qualification Plan

To achieve BQP goals, participants submit information and receive FDA feedback through a structured, three-phase process: (1) letter of intent (LOI), (2) qualification package (QP), and (3) full qualification package (FQP) (Fig. 1).

In the final stage, the Center for Drug Evaluation and Research (CDER) qualifies the biomarker for the defined context of use (COU) in any drug development program to support regulatory decision-making. The phased and collaborative BQP process is intended to facilitate qualification of regulatory-grade biomarkers by providing an avenue for development outside the context of a single clinical development program, offering feedback at each submission, and the ability to seek advice through meetings with BQP staff.

Previous analyses have examined participation in and the impact of other drug development tool (DDT) qualification programs, such as the clinical outcome assessment (COA) qualification program, and characterized the stakeholder groups engaged in the BQP [4, 5]. Here, we assess the use of the BQP over the past eight years to evaluate whether the program facilitated the qualification of novel biomarkers as intended, including those for use as early measures of treatment efficacy (i.e., surrogate endpoints).

## Materials & Methods

We collected data on biomarker qualification projects from the CDER and Center for Biologics Evaluation and Research (CBER) Drug Development Tool (DDT) Qualification Project Search database [6].

### Project Characteristics and Submission Progress

For each project, we collected information on the latest submission stage (LOI, QP, FQP), latest submission status as of July 1, 2025 (accept, not accept, qualified, submitted, withdrawn/rescinded), and project characteristics (biomarker category, biomarker type, measure of [disease or condition, drug response/effect of exposure], and surrogate endpoint [yes/no]). Where available, we also recorded the project’s date of acceptance into the program, dates of each submission (LOI, QP, FQP), and the date of the FDA’s determination for each submission. Although not a formal stage of the BQP submission process, dates of submission and determination for Section 507 Transition Plans were also collected for projects initiated under the FDA’s legacy process for drug development tool qualification.

### Submission Timelines

For projects with available submission and determination dates, we calculated LOI review time (months from LOI submission to FDA determination) and QP review time (months from QP submission to FDA determination). For projects that submitted a qualification plan, we also calculated QP development time (months from FDA determination for the LOI to QP submission). For biomarker projects that reached full qualification, the database included only the date(s) of qualification; therefore, time to qualification could not be calculated. In some cases, multiple submission and determination dates were reported (e.g., an initial QP submission that received a “not accept”, followed by a QP resubmission, and an FDA “accept” determination). In these instances, we used the last reported submission and determination date.

## Results

As of July 1, 2025, the FDA’s DDT Qualification Project Database listed 99 projects under the biomarker qualification program. Sixty-one of these projects (62%) were accepted into the program.

### Characteristics of Accepted Biomarker Qualification Projects

Accepted projects represented a diverse range of biomarkers with varying methods of assessment and intended uses (Table 1).

**Table 1:** Characteristics and latest submission stage of accepted biomarker qualification projects. The biomarker qualification program (BQP) has had broad application with accepted projects focusing on a variety of biomarker types, categories, and measures

	Total	507 Transition Plan^1^	Stage 1: LOI	Stage 2: QP	Stage 3: FQP
Total	61 (4 wd)	5 (1 wd)	30 (1 wd)	18 (2 wd)	8
Biomarker category					
*Diagnostic*	13 (1 wd)		8	4 (1 wd)	1
*Monitoring*^2^	5		4		1
*PD Response*^2^	12 (1 wd)	2	4	6 (1 wd)	
*Prognostic*	12		8	2	2
*Safety*	18 (2 wd)	3 (1 wd)	5 (1 wd)	6	4
*Susceptibility/Risk*	2		2		
Measure of					
*Disease or Condition*	30 (1 wd)		19	7 (1 wd)	4
*Drug Response/Effect of Exposure*	30 (3 wd)	5 (1 wd)	10 (1 wd)	11 (1 wd)	4
*Not Specified*	1		1		
Biomarker type					
*Composite*	1		1		
*Histologic*	4	1	2	1	
*Molecular*	28 (1 wd)	2 (1 wd)	11	8	7
*Physiologic Characteristics*	4 (1 wd)	1	3 (1 wd)		
*Radiologic/Imaging*	24 (2 wd)	1	13	9 (2 wd)	1
Surrogate endpoint					
*Yes*	5	1		4	
*No*	56 (4 wd)	4 (1 wd)	30 (1 wd)	14 (2 wd)	8

^1^507 Transition is not a formal phase of the DDT qualification process established under Section 507 of the 21st Century Cures Act; it serves as a transition phase for projects initiated under the legacy process for biomarker qualification (pre-2017).

^2^One project is categorized as both a PD Response and a Monitoring biomarker and is included in the total projects for each category. LOI, Letter of Intent; QP, Qualification Plan; FQP, Full Qualification Package; wd, Withdrawn/Rescinded; PD, pharmacodynamic.

Safety biomarkers were most common (18/61, 30%) followed by diagnostic biomarkers (13/61, 21%), pharmacodynamic (PD) response biomarkers (12/61, 20%), and prognostic biomarkers (12/16, 20%). For the method of assessment (i.e., biomarker type), molecular biomarkers (28/61, 46%) and radiologic/imaging biomarkers (24/61, 39%) were most frequently explored. Projects were evenly split between biomarkers intended to measure a disease or condition (*n* = 30/61, 49%) and those intended to measure drug response/effect of exposure (*n* = 30/61, 49%), with one project uncategorized. While about half of accepted projects included biomarkers considered to be measures of drug response/effect of exposure, only a small fraction included biomarkers intended to be used as surrogate endpoints (5/61, 8%).

### Submission Stage and Status

**Fig. 2:**
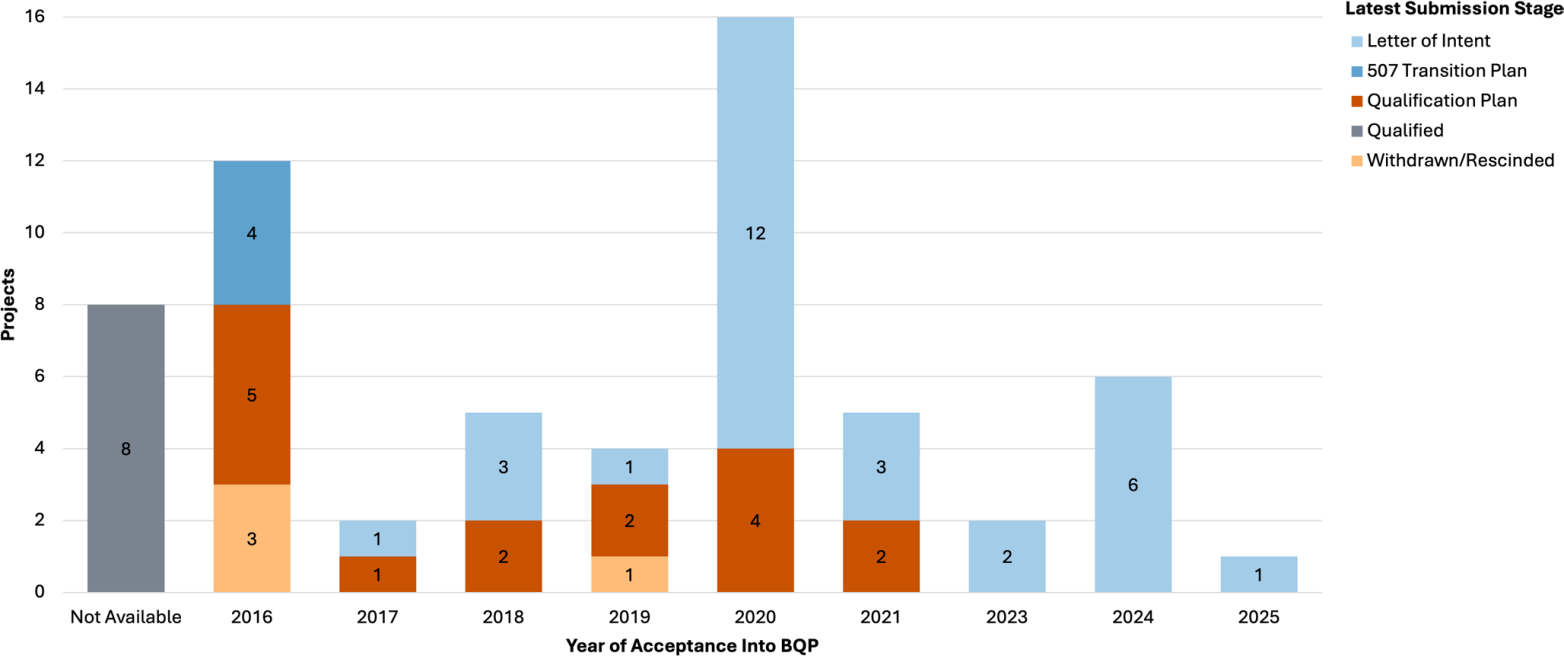
Latest submission stage by year of acceptance into the biomarker qualification program. Note: “Not Available” includes qualified biomarkers for which acceptance and submission dates were not reported in the database. BQP, Biomarker Qualification Program

About half of all accepted projects (30/61, 49%) have not progressed past the initial LOI stage of the BQP process, and four projects were withdrawn or rescinded after acceptance. Many projects that remain at the LOI stage and have not been withdrawn or rescinded are likely still developing a qualification plan. Only eight biomarkers were qualified through the program, and seven of these were qualified before the *21st Century Cures Act* was enacted in 2016 under the FDA’s legacy biomarker qualification process (Fig. 2).

The most recent qualification was granted in 2018. Among the biomarkers that achieved qualification, most were safety biomarkers (4/8, 50%) and predominantly used molecular methods for assessment (7/8, 88%). Importantly, no biomarker projects that included a surrogate endpoint have reached qualification; however, 4/5 of these projects submitted a qualification plan, of which 3 were accepted by the FDA.

### Submission Timelines

Median LOI and QP review times exceeded the time frames specified in the November 2020 Final FDA guidance document titled “Qualification Process for Drug Development Tools”^1^ [7]. Among the 43 projects with LOI submission and determination dates reported, LOI reviews took a median of 6 months—twice as long as the 3-month target time frame outlined in the guidance. Notably, most (31/43, 72.1%) of these projects were accepted into the BQP pre-final guidance. However, among the 12 projects submitted since the finalization of this guidance, LOI reviews have continued to exceed expected timelines, taking a median of 13.4 months (compared to 5.1 months for the 31 projects submitted pre-guidance). For the 13 projects with available QP submission and determination dates, 10 were submitted post-final guidance. Overall, QP reviews took a median of 14 months—7 months longer than the guidance-specified time frame. For post-guidance submissions, QP reviews took a median of 11.9 months.

**Fig. 3:**
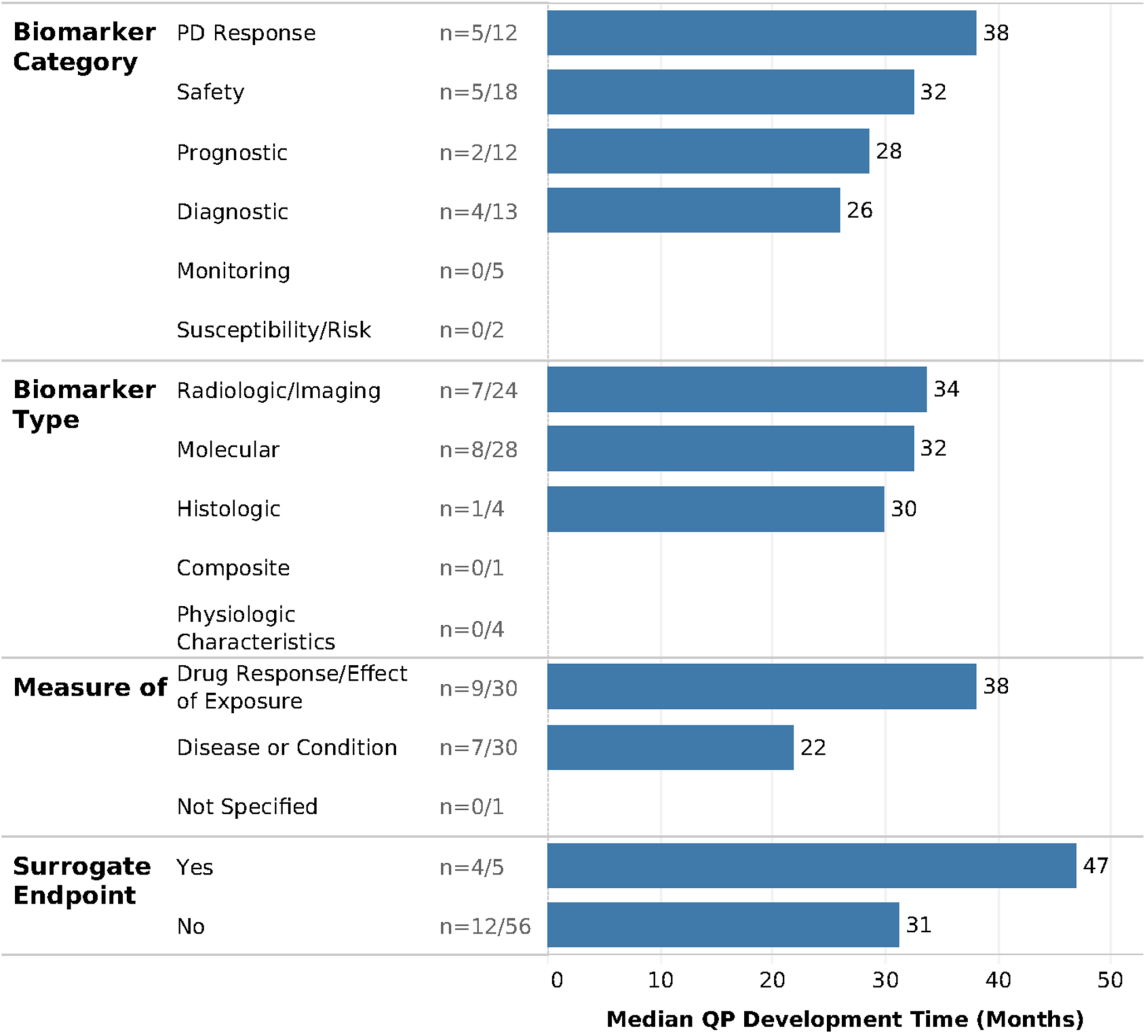
Qualification plan development time by characteristics of biomarker qualification projects. Qualification plan development timelines varied across projects depending on the biomarker’s characteristics, with surrogate endpoint biomarkers requiring 16 months longer for QP development than other biomarker projects. PD, pharmacodynamic; QP, Qualification Plan

QP development time was also calculated for the 16 projects with LOI determination and QP submission dates available. For these projects, it took a median of 32 months (2.7 years) to develop and submit a qualification plan. QP development timelines varied depending on the project characteristics. PD response biomarkers (*n* = 6) and biomarkers assessing drug response/effect of exposure (*n* = 11) had longer QP development timelines, with a median of 38 months (3.2 years) from LOI acceptance to QP submission, compared to other biomarker categories and measures (Fig. 3).

Projects across the various biomarker types (molecular, radiologic/imaging, and histologic) had similar QP development times. Qualification plans for surrogate endpoints (*n* = 4) took the longest to develop overall, with a median of 47 months (3.9 years), reflecting the extensive requirements to validate a novel surrogate endpoint.

## Discussion

The formalization of the DDT qualification process, including the process for qualifying biomarkers under the BQP, created a transparent, structured process for collaboration and information exchange to optimize the development and validation of novel biomarkers. However, in the eight years since the *21st Century Cures Act* formalized this framework, the program’s overall impact appears limited. Only eight biomarker projects have reached full qualification, and all were reviewed under the legacy qualification process before the Section 507 process was established by the *21st Century Cures Act*. For ongoing projects, reviews often exceed expected timelines, and about half have not progressed beyond initial LOI acceptance. These trends have continued for the 12 projects submitted in the five years since the November 2020 final guidance on the qualification framework; however, additional time may be needed to assess whether the guidance has an impact on QP development times, or if these trends continue.

The BQP appears to be more impactful for certain biomarker categories, particularly safety biomarkers, which account for roughly one-third of accepted projects and four of the eight qualified biomarkers. In contrast, despite stakeholder interest in developing novel biomarkers to measure treatment efficacy, the program has seen very limited use for biomarkers intended as surrogate endpoints. It is important to understand what drives the lack of engagement and whether the current framework is ill-suited for qualifying these more complex biomarkers. Surrogate endpoint projects advance more slowly, with QP development requiring nearly 4 years–16 months longer than other biomarkers. The BQP lacks a funding mechanism, and there is limited guidance on opportunities for iterative FDA interaction throughout the process. Many factors can lead to delays or a failure to reach qualification, and targeted engagement and collaboration can help to efficiently plan, navigate challenges, and advance surrogate endpoint qualification. An optimized program could provide timely advice to help researchers identify and address potential challenges and, if they are unable to be overcome, facilitate more efficient withdrawals from the qualification program.

Given the promise of surrogate endpoints to expedite treatment evaluations—and the extensive evidence required for them to be deemed reliable for regulatory decision-making—targeted enhancements and dedicated funding for the BQP, potentially supported by allocating PDUFA resources to divisions participating in the review of a given qualification plan, could enable more timely, structured engagement with specific FDA therapeutic area experts. Additional resources to support the divisions overseeing biomarker qualification should be accompanied by expected timelines for interactions such as meeting requests and conduct, feedback deadlines, and review periods. Such an approach would help embed BQP review into the established workflow associated with traditional user-fee-supported application review.

The Rare Disease Endpoint Advancement (RDEA) Pilot program, initiated under PDUFA VII, demonstrates a model for supporting the development of novel endpoints. However, RDEA is limited to endpoints that are associated with an active investigational new drug application (IND) [8]. Unlike the BQP, participating in RDEA may not enable qualification of the endpoint for broader use in any development program within a specific COU. While this approach may be sufficient for enabling endpoint development for rare diseases, it is less suited for endpoints with broader applicability, such as those relevant across multiple oncology development programs (e.g., circulating tumor DNA [ctDNA], minimal residual disease [MRD], pathologic complete response [pCR]), which typically require pooled data from multiple studies and sponsors [9–15]. In these instances where the biomarker could be applied to numerous future development programs, encouraging development and validation across multiple stakeholders and sponsors would be important to not limit use of the biomarker to a single program or sponsor’s portfolio. While the BQP has been suitable for developing certain types of biomarkers (e.g., safety biomarkers), for novel endpoints and response biomarkers, particularly surrogate endpoints, which generally face a higher evidentiary bar for regulatory acceptance, funding and additional opportunities for engagement could provide the targeted and collaborative framework necessary to efficiently advance their development.

## Conclusion

Enhancements to the existing BQP to support the development of novel biomarkers and endpoints for assessing treatment response not tied to a single IND or development program could help better prioritize and advance collaborative efforts to validate these tools. A more flexible framework that enables collaborative, pre-competitive evidence generation and sustained regulatory engagement would be needed for such biomarkers to achieve validation. Building on the phased structure of the BQP, the framework could incorporate iterative submissions and feedback touchpoints on data and validation plans, while offering enhanced opportunities for engagement between FDA and stakeholders to prioritize high-impact measures and guide their advancement. Creating this type of pathway could unlock the potential of novel response biomarkers to expedite drug development and improve patient access to effective therapies.

## Data Availability

No datasets were generated or analysed during the current study.

## Footnotes

1 The FDA guidance document specifies reviews are, “The time taken to review a submission once FDA has deemed it reviewable and a memorandum notifying the requestor of receipt of a reviewable submission has been sent to the requestor. For LOI, QP, and FQP submissions, the time frames are targeted to be completed within 3, 6, and 10 months, respectively, from the date on the reviewable memorandum”.
